# A meta-analysis on decomposition quantifies afterlife effects of plant diversity as a global change driver

**DOI:** 10.1038/s41467-020-18296-w

**Published:** 2020-09-11

**Authors:** Akira S. Mori, J. Hans C. Cornelissen, Saori Fujii, Kei-ichi Okada, Forest Isbell

**Affiliations:** 1 Graduate School of Environment and Information Sciences, Yokohama National University, 79-7 Tokiwadai, Hodogaya, Yokohama, 240-8501 Japan; 2 Department of Ecological Science, Faculty of Science, Vrije Universiteit, De Boelelaan 1085, 1081 HV Amsterdam, The Netherlands; 3 Forestry and Forest Products Research Institute, Tsukuba, Ibaraki 305-8687 Japan; 4 Faculty of Bioindustry, Tokyo University of Agriculture, 196 Yasaka, Abashiri, Hokkaido 099-2493 Japan; 5 Department of Ecology, Evolution and Behavior, University of Minnesota, St. Paul, MN 55108 USA

**Keywords:** Biodiversity, Community ecology, Ecosystem ecology, Biodiversity

## Abstract

Biodiversity loss can alter ecosystem functioning; however, it remains unclear how it alters decomposition—a critical component of biogeochemical cycles in the biosphere. Here, we provide a global-scale meta-analysis to quantify how changes in the diversity of organic matter derived from plants (i.e. litter) affect rates of decomposition. We find that the after-life effects of diversity were significant, and of substantial magnitude, in forests, grasslands, and wetlands. Changes in plant diversity could alter decomposition rates by as much as climate change is projected to alter them. Specifically, diversifying plant litter from mono- to mixed-species increases decomposition rate by 34.7% in forests worldwide, which is comparable in magnitude to the 13.6–26.4% increase in decomposition rates that is projected to occur over the next 50 years in response to climate warming. Thus, biodiversity changes cannot be solely viewed as a response to human influence, such as climate change, but could also be a non-negligible driver of future changes in biogeochemical cycles and climate feedbacks on Earth.

## Abstract (web summary)

There is evidence that reducing plant litter diversity may slow litter decomposition rate. Here, Mori and colleagues perform a global meta-analysis of litter-bag studies to show that mixed-species litter assemblages decompose faster than monospecific assemblages, with a magnitude comparable to the predicted effect of climate warming.

## Introduction

An increasing body of knowledge considers how people and our society are influential to and dependent on ecosystems and biodiversity therein^1^. The biodiversity loss crisis^2^ has led to the foundation of many local and international frameworks including the Convention on Biological Diversity and the Intergovernmental Science-Policy Platform on Biodiversity and Ecosystem Services to tackle this crisis^3,4^. Along with these schemes primarily aimed at halting the loss of species and conserving organisms in ecosystems, a series of evidence has also accumulated for how biodiversity is fundamental to support ecosystem functions and thus services for humanity^1,5–7^. This evidence stems from local-scale manipulations of biodiversity and the related theoretical works^5,8^. Especially, there has been a primary focus on the diversity effects of plants as primary producers on the flows and stock of the organic matter and energy in ecosystems^5^. Currently, such evidence is becoming even more important at the scales that are relevant to policy-making^1,9^. Hundreds of previous studies have considered how the diversity of living primary producers affects productivity and decomposition^10,11^. However, there remains a lack of synthesis of the studies on how and how much litter decomposition depends on plant diversity (but see^12^). Consequently, there is less confidence in how the after-life effects of plant diversity^13,14^ determine biogeochemical cycles and climate feedbacks on Earth, emphasizing the need to have a comprehensive synthesis by gathering broad information from ecosystems in different biomes and climates.

Along with primary production, decomposition of organic matter is a key process in which biota play a fundamental role^15^. In this regard, increasing diversity in terms of plant litter species can promote the decomposition process^16,17^. Possible mechanisms underlying such biodiversity effects include nutrient transfer between different litter species with different decomposability^14^, altered decomposer activity by specific litter traits^18^, and positive feedback of decomposers resulting from the divergence in resource use and habitat availability^19^. While individual studies have carefully disentangled possible mechanisms within sites, there is limited synthesis of directions and effect sizes of decomposition response to diversity available to date (but see^12^). Advancing our understanding on these responses is important, because of the ongoing anthropogenic pressures on the biosphere; for instance, habitat simplification and degradation^20^. If vegetation is altered and plant species diversity is simplified, as is often the case in the conversion to monocultures, decomposer communities living in them and surrounding environments such as streams can also be simplified due to substrate and habitat simplification^21,22^; such biotic homogenization could have substantial consequences on ecosystem processes^23^, including decomposition^24,25^. Another threat is climate change. It has become increasingly clear that climate change is altering decomposition rates^26^. Because decomposition is a primary determinant of carbon and nutrient dynamics in the biosphere^27^, alteration of the after-life effects of diversity should have important consequences for global biogeochemical cycles and climate feedbacks. It remains unclear, however, whether models of vegetation dynamics and biogeochemical cycles should also incorporate influences of biodiversity^28^. It is therefore important to synthesize the knowledge of how plant diversity could influence decomposition processes through the diversity of litter, and to compare the magnitudes of these diversity effects to those of other regulators of decomposition such as climate.

**Fig. 1:**
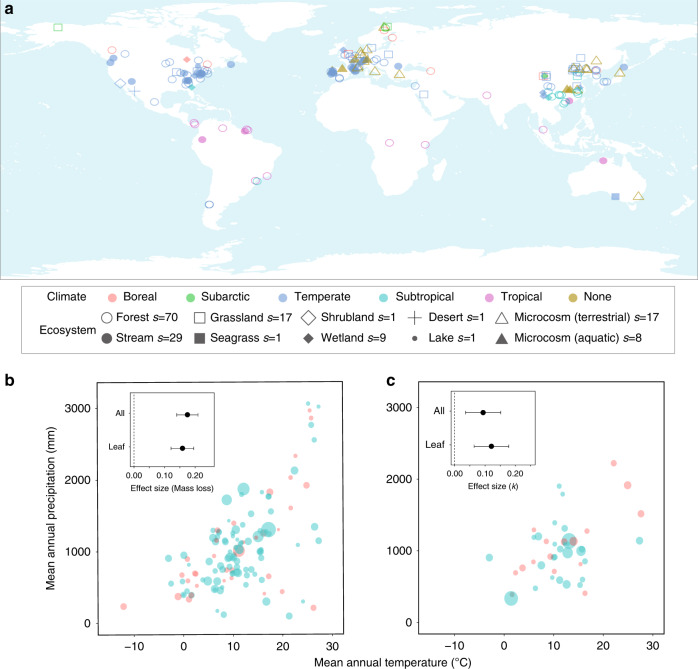
Overview of the dataset. **a** A map showing the geographical distributions of the studies for the diversity effect (species-mixing effect) on plant litter decomposition. Different shapes and colours of symbols represent different ecosystem types and climatic zones. The number of studies (*s*) is shown for each ecosystem type; note that some studies simultaneously conducted multiple experiments in different ecosystem types and climate regions. **b**, **c** Mean annual temperature and precipitation are shown for each of the studies that evaluated mass loss (131 studies) and/or the decomposition rate constant *k* (45 studies). Symbols represent each study, where the size of circles is proportional to the within-study mean effect size of Hedges’ *d* (based on mass loss and *k* as a measure of decomposition rate) and their colours if the mean is positive (blue) or negative (red). Small inset figures are the effect sizes (**b** mass loss; **c** the constant *k*), analyzed for all litter types and for leaf litter. Closed circles and error bars represent means and the 95% confidence intervals, respectively. A multilevel mixed effects meta-regression was used to account for a nested structure of the dataset. Vertical dotted lines are to indicate the effect size of zero.

Here, we provide a global-scale meta-analysis for the after-life effects of plant diversity (species-mixing effects) on litter decomposition by gathering relevant data from in situ and ex situ litter-bag experiments (Fig. 1a). We primarily focus on mass loss after a given litter incubation period as a measure of decomposition rate because this measure was most often reported in previous studies (131 studies). In addition, the decomposition rate constant *k* (negative exponent of the curve describing litter mass remaining over time) is used, as it integrates mass loss over more than one time interval (45 studies). Importantly, the rich body of evidence for the after-life effects of diversity allows us to see how these effects could change between different litter types (e.g., leaves and twigs), biomes, and climatic regions. Given the increasing interest in assessing the relative contributions of these local factors to decomposition^29^, a global synthesis is timely. After synthesizing effects of litter diversity on decomposition rates, we compare the magnitude of these effect sizes to those of climate change to assess how much changes in biodiversity could alter decomposition compared with changes in decomposition that have been projected to occur over the next 50 years in response to climate warming (based on the 2.6 and 8.5 scenarios of representative concentration pathways of CO_2_; RCP 2.6 and 8.5, respectively).

Here, we show that the after-life effects of plant diversity to increase the rates of decomposition are significant across different biomes. When focused on forests worldwide, their effect sizes are comparable to the possible climate change impacts projected by 2070s. We thus emphasize that incorporating the underexplored roles of biodiversity into the assessment of future changes in the biogeochemical cycles and climate feedbacks is critical in this era of global environmental changes.

## Results and discussion

### After-life effects of plant diversity

Our dataset is comprehensive by covering the broad range of climatic regions and extensive in considering many possible comparisons for decomposition rate between mixed and mono-species litter in different biomes (Fig. 1a). Our dataset for mass loss measurement and estimation of the constant *k* comprises in total 6535 comparisons across 1949 different treatments reported in 131 studies (Fig. 1b) and 1423 comparisons across 504 different treatments reported in 45 studies (Fig. 1c), respectively. Based on a multilevel random effects meta-analysis^30^ that can account for differences between studies and treatments, we found that, across all studies, increasing plant diversity (species-mixing effects) significantly increased the rate of decomposition (Fig. 1b, c; *p* < 0.0001 and *p* < 0.01 for mass loss and the constant *k*, respectively). We confirmed little publication bias due to non-significant results not being reported (Supplementary Fig. 1). We additionally checked for the cases where we allowed one data point per treatment and found little difference between the main result and results from this confirmation procedure, suggesting that the results are little affected by autocorrelation or non-independence (Supplementary Fig. 2). When we focused on leaf litter, i.e., excluding the relatively few studies with other types of plant litter such as root and twig litter, moss, and macrophytes, we found very similar results (Fig. 1b, c; *p* < 0.0001 for both mass loss and the constant *k*). Thus, we conclude that the after-life effects of plant diversity in promoting decomposition predominate across biomes.

**Fig. 2:**
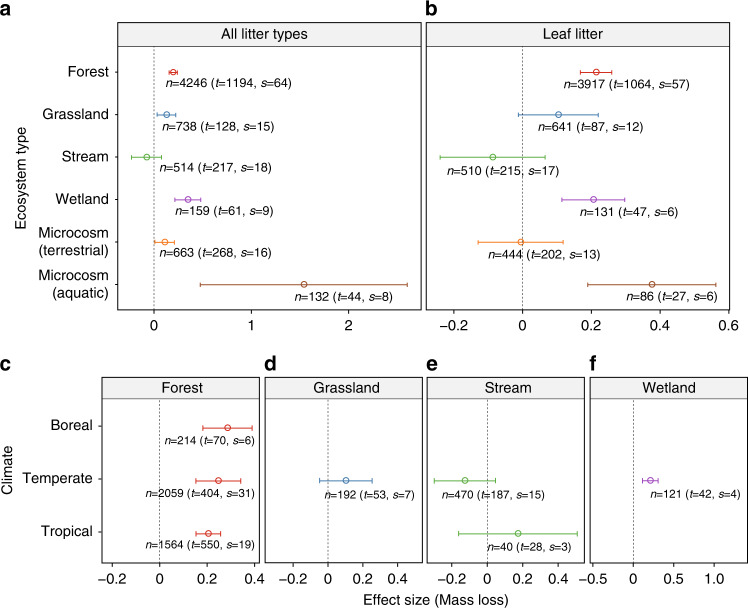
The results for the effect size of the diversity effect (species-mixing effect) on plant litter decomposition. The results are based on mass loss as a measure of decomposition rate. Note that ranges of *x*-axis differ among the panels **a**–**f**. **a** Analyzed by ecosystem types for all litter types; **b** by ecosystem types for leaf litter; **c**–**f** by ecosystem types and climatic zones for leaf litter. Open circles and error bars represent means and the 95% confidence intervals, respectively. Number of comparisons (*n*), treatments (*t*), and studies (*s*) are shown. Positive values of the effect size indicate faster decomposition in mixed-species litter than mono-species litter. Vertical dotted lines are to indicate the effect size of zero. Mixed-effect meta-regression showed significant differences in the effect sizes when analyzed with ecosystem types (*Q*_*M*_ = 52.64, d.f. = 5, *p* < 0.0001), climate regions (*Q*_M_ = 13.30, d.f. = 5, *p* = 0.02), and both of them together (*Q*_M_ = 69.69, d.f. = 10, *p* < 0.0001) as a moderator variable(s). For a comparison by climate zones, tropical and subtropical biomes were assigned as tropics to ensure the number of studies required for the analysis. Results for data types with three and more studies are shown.

When analyzed by ecosystem types, we found significant positive litter species-mixing effects for most biomes except streams (Fig. 2a). We also conducted the same analysis for subsets of the data that focused on leaf litter (Fig. 2b) and excluded comparisons with no identity information available for litter species (Supplementary Fig. 3); we found that the overall trends were mostly identical. Note that we found no litter diversity effect in streams (Fig. 2a, b), which is contrasting to previous syntheses^14,25^ and an important issue to be explored. Here, we speculate that active transfer of nutrients among litter of different species by decomposers, a key mechanism driving the litter-mixing effects^31^, could be prevented by water flow^17^ or weakened due to dissolved nutrients in water^32^. Nutrient enrichment in stream water, which can suppress the diversity effects^33^, could occur especially at large spatial scales^32^. Other abiotic factors such as sediment disturbance also predominate in large-sized downstream, and thus biotic processes such as the mixing effects on litter breakdown can be only significant in low-order, small streams^25^. Our synthesis could not fully account for such context dependency. Considering these possibilities and positive diversity effects in other aquatic ecosystems found in this study (Fig. 2a, b), we call a need for further efforts, including assessments for the involvement of decomposers^17,19,34,35^, different environmental conditions^25,33,35,36^, and functional traits of plants^18,25,32,37^, which can determine the direction and magnitude of litter diversity effects. Notwithstanding such uncertainties and potential interactions between abiotic and biotic factors, the present results give an important quantification for the roles of biodiversity on decomposition rates not only under controlled *ex situ* conditions but also under the heterogeneous environments in nature.

Interestingly, when the data were further partitioned by ecosystem types and by climate, we found between-biome differences in the species-mixing effects (Fig. 2c–f). The comparison from the tropical to the boreal biome was only possible for forest studies, where these effects have been most often studied. In forests, we found that decomposition fostered by species-mixing effects tended to be more prominent in colder biomes (*Q*_M_ = 8.69, *p* = 0.069), further suggesting the important interactions between biodiversity and the environment. A study that focused on the roles of tree diversity on productivity in forests^38^ showed that potential mechanisms underpinning the biodiversity-ecosystem functioning relationships could shift from subtropical to boreal forests, because of the increased role of the complementarity effects^39^ with increasing the environmental harshness; a finding in line with the stress gradient hypothesis^40^. Considering the potential importance of the complementarity effects in mixed-species litter decomposition^14,29^, the present finding for the prominent diversity–decomposition relationships in colder forest could also be related to similar mechanisms. We thus advocate further systematic cross-site studies to carefully disentangle the relative importance between climate and biotic controls on decomposition^41–43^.

### Climate-equivalency of the diversity effects

Like climate change, biodiversity change can also be considered as one of the strongest drivers of global change^44,45^. Thus, we compared the magnitudes of the after-life effects of plant diversity on litter decomposition rates with possible scenarios of climate change (two scenarios of representative concentration pathways used in the 5th Climate Model Intercomparison Project; CMIP5 RCP 2.6 and 8.5) by the 2070s. Note that litter decomposition is primarily controlled by three factors; climate (e.g., temperature and moisture), decomposers, and litter traits^41,46–48^ and this is also the case for mixed-species litter^31,49^. We thus removed the influences of decomposers and litter traits on the process and obtained a standardized equation of the climate–decomposition relationships; this was realized through using a dataset of a full reciprocal litter transplant experiment that used various plant species differing in litter traits and covered a broad range of biomes from tropical to subarctic forests^50^. Briefly, the equation was used to estimate by how much temperature or precipitation would need to change in order to alter litter decomposition as much as it would be altered by changes in plant diversity in forest biomes. These estimates of climate-equivalency of the diversity effects were used to estimate potential increases in the decomposition rate in the study locations of the forest biomes under a scenario of litter diversification from mono- to mixed-species (see “Methods”).

**Fig. 3:**
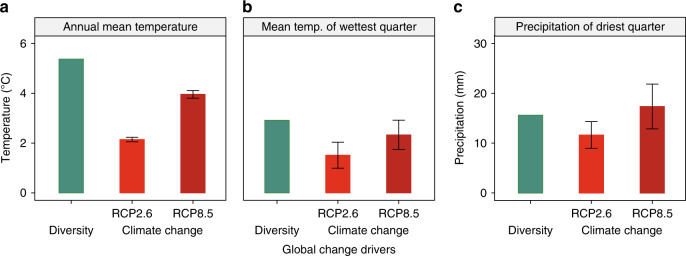
The effects of different global change drivers (biodiversity and climate change) on plant litter decomposition in forest biomes. **a** Comparison of the diversity effects (diversifying from mono- to multiple litter species) with the climatic effects based on annual mean temperature; **b** based on mean temperature of the wettest quarter; **c** precipitation of based on the driest quarter. The climatic effects were estimated using the dataset of a full reciprocal transplant experiment^50^, which made it possible to remove the influences of plant litter traits and decomposers on decomposition rate; the effect size of the diversity effects (species-mixing effects) was estimated for the dataset of the present meta-analysis (for 57 forest studies that used leaf litter) and, based on temperature/precipitation required to change the effect size to the same magnitude as the diversity effects, the climate-equivalency of the diversity effects was estimated (turquoise bars). The two future projections (in 2070s) of climate changes (based on the CMIP5 RCP 2.6 and 8.5 scenario; means and standard deviations are shown as thick red bars and thin black error bars, respectively) are shown for the study locations of the present meta-analysis. See Table S1 for additional analyses using different subsets of the data.

**Fig. 4:**
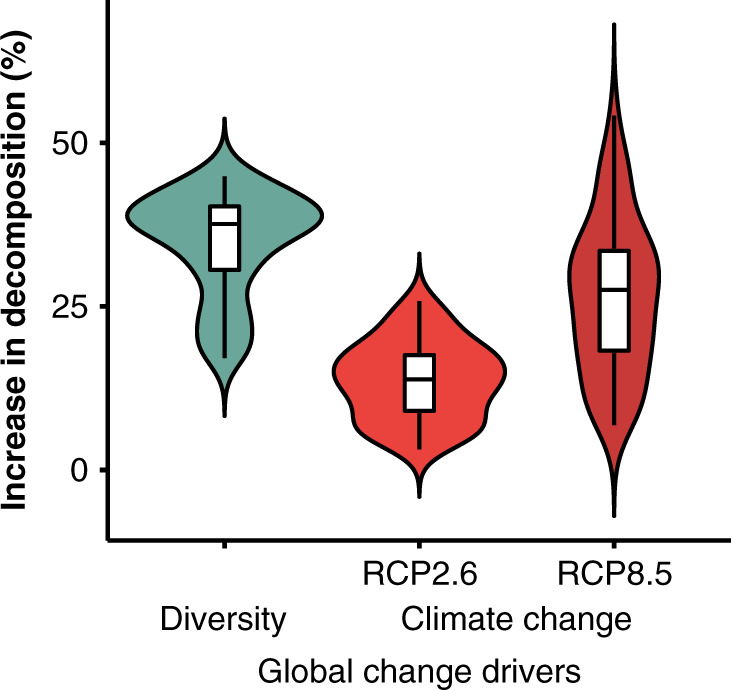
Potential changes in decomposition rates due to global change drivers. Violin plots and boxplots showing potential increases (%) in the process of decomposition (mass loss) resulting from diversity change or climatic warming, estimated for the study locations of the present meta-analysis (57 studies in the forest biomes). These estimates were based on the climate-equivalency of the diversity effects (Fig. 3a) and the standardized climate–decomposition relationships. Diversifying plant litter from mono- to mixed-species could increase the decomposition by 34.7% (mean). Based on the future projections of climate changes in these study locations (increases in annual mean temperature; CMIP5 RCP 2.6 and 8.5; Fig. 3a), decomposition could be increased by 13.6% and 26.4% (means), respectively. The violin plots represent the kernel probability density of the values. The boxplots denote the interquartile range (box) and median (centre line) of the values.

We found that the after-life effects of plant diversity on decomposition rate are overall comparable in magnitude to the effects of future climate change projections (Fig. 3). Specifically, diversifying plant litter from mono- to mixed-species could increase the decomposition by 34.7% in the forest biomes, which is substantial and comparable to projected increases in forest litter decomposition due to climatic warming (13.6% and 26.4%, based on the scenarios of CMIP5 RCP 2.6 and 8.5, respectively; Fig. 4) in these locations. The effects of plant diversity on decomposition have previously been assumed to be less strong than those on biomass production^10,13,51^, perhaps partly because of limited quantitative syntheses for the former. We now fill this knowledge gap and show that, even compared with well-studied effects of plant diversity on biomass production^10,52^, the after-life effects of plant diversity on decomposition are substantial in magnitude. These counter-balancing effects of changes in plant diversity on biomass production and decomposition have been best studied in different systems (grasslands and forests, respectively), and thus it remains difficult to quantify the net effect of changes in plant diversity on the global carbon cycle. Nevertheless, our results importantly demonstrate the potential magnitude of biodiversity changes to alter the biogeochemical cycles and climate feedbacks on the Earth.

### Implications

We have synthesized the knowledge on how plant diversity could influence decomposition processes through the diversification of litter. However, caveats are surely due in this study. First, most of the available data are for monocultures and mixtures of two or three species, which limits our ability to comment on communities with more species. However, it is unlikely to have many plant species interacting in the small surface area where individual litter accumulates^53^ (such as that of a litter-bag), so that this should not be a serious issue. Second, it is likely that the biodiversity–ecosystem functioning relationships change with time and differ between systems^25,54,55^, as also found in the present dataset (Supplementary Fig. 4) and elsewhere^12^, emphasizing the context-dependency of the after-life effects of diversity. Especially, our results might be seemingly different from a recent synthesis^12^, which concluded that litter species-mixing effects on decomposition were negligible; however, the study, in fact, found a significant positive effect when analyzed based on sample sizes and sampling variances of the effect sizes (as conducted in our study). Thereby the two syntheses are not necessarily contradictory. Third, it was not possible to assess how another facet of biodiversity, i.e., decomposers^16,17,19,42^, the diversity of which is fundamental to ecosystem functions^56,57^, was involved in the relationships of litter diversity-decomposition, emphasizing the need for further studies to have a broad picture of the roles of biodiversity in ecosystems. Last, our equations for the climate–decomposition relationships (also see Supplementary Table 1; Supplementary Fig. 5) could not fully capture possibilities for other climatic gradients. We thus emphasize that further studies will be needed to fully understand the roles of litter diversity in diverse plant communities.

Notwithstanding these uncertainties, our study has important implications. The existing modelling approaches for considering biogeochemical cycles take account of many differences in biophysical attributes (e.g., albedo) between different land-cover, vegetation and ecosystem types; however, they are not so advanced that they incorporate possible differences in biotic interactions^28^ including those we have shown here. Specifically, despite the decreasing trend of global forested areas, the areas of monoculture plantation forests, *so-called* ‘Green desert’, are increasingly spreading in many regions^58,59^. By remote-sensing, it is feasible to identify species-rich and poor forests (i.e., canopy tree species), which are currently rather ignored despite their possible differences in the biogeochemical cycles supported by biodiversity^60^. The present estimates for the roles of species mixture in enhancing the processes of terrestrial litter decomposition, which we found non-negligible, would help improve these assessments in these contrasting vegetation compositions. That is, the present study can inform the land-surface and the biogeochemical models to incorporate the unexplored roles of biotic interactions in determining carbon and nutrients flow through decomposer subsystems, which could be critical for improving their future projections in this era of global environmental changes.

## Methods

We primarily relied on the “*tidyverse*”^61^, “*metafor*”^30^, “*lmerTest*”^62^, “*effects*”^63^, “*maptools*”^64^, “*sf*”^65^, and “*raster*”^66^ packages of the R software^67^ for organizing and analyzing data, and visualizing the results.

### Data assembly

To support the use of meta-analysis to quantify the effects of species richness on plant litter decomposition (i.e., species-mixing effects), we searched the literature using the ISI Web of Science (WoS) database (https://clarivate.com/products/web-of-science/; up to and including December 2018). We used a combination of “decomposition” AND “litter”. These keywords matched 12,278 publications. We then reduced this list of the literature with the keywords of “mix* litter,” OR “litter diversity,” OR “litter mix*,” OR “litter species diversity,” OR “litter species richness,” OR “species mix*,” OR “mix* plant litter,” OR “multi* species litter,” OR “mixing effect*,” resulting in 416 publications. We also searched the literature using Scopus (https://www.scopus.com/home.uri) and Google Scholar (https://scholar.google.ca/) and the latter set of keywords, resulting in 765 publications. An anonymous expert identified 15 additional publications. We decided not to include reports in the grey literature among the papers we found, as we could not verify whether, as in all WoS-listed papers, the papers had undergone independent peer review as a quality check. We read through the papers carefully to select those that focused on quantifying the effects of litter diversity on decomposition rates based on a litter-bag experiment using both mixed- and mono-species litter. We focused on the publications that reported values of either mass loss (or mass remaining) or a decomposition rate constant *k* that quantified the decomposition rate. Specifically, some papers for litter-mixing effects reported only for the additive effects of mixture and had no reports on mass loss or the constant *k*. In some cases, no sample size nor standard deviations/errors were reported. Mass loss or standard deviations/errors values were reported only for mono-species or multi-species litter. These publications were not included in the present analyses. In cases that had no report on means but instead showed median values, we estimated means and standard errors based on individual data points or percentile values of boxplots. Note that, standard deviations/errors were often invisible because they were too small to read and thus hidden by their data points; in this case, a conservative approach was adopted by using possible maximum values of deviations/errors (i.e., the size of these symbols). Based on these criteria, we extracted information required for our meta-analysis (see below) from a total of 151 studies (Supplementary Data 1). Out of these 151 studies, 131 and 45 studies measured mass loss and the decomposition rate constant *k*, respectively. Also see the PRISMA work flow diagram (Supplementary Fig. 6).

For the selected studies, we identified the sets of comparisons between mixed- and mono-species litter bags in each publication. That is, a single study could have multiple treatments that focused on different litter substrates, using different mesh sizes for litter bags, having multiple experiments in different locations, changing abiotic conditions, and so on. The decomposition rate should be comparable in a given treatment under the same set of environmental conditions except for differences caused by a different number of plant species. Even for a given treatment, decomposition rates were often reported multiple times in the literature; in such cases, we only included comparisons for litter retrieved at the same time (i.e., after the same incubation period). Note that in a given treatment, the same mono-species litter could be used for multiple comparisons; for instance, mass loss from a litter bag that contained a three-species mixture (species A, B, and C simultaneously) can be compared with the mass loss in three different mono-species litter bags (only species A, B, or C). This could lead to issues related to pseudo replication, which we considered based on a multilevel random effects meta-analysis (see Data analyses). As a result, we identified 6535 comparisons (across 1949 treatments from the 131 studies) for the values of mass loss and 1423 comparisons (across 504 treatments from the 45 studies) for values of the decomposition rate constant *k*.

After identifying the sets of comparisons, we extracted the sample size (number of litter-bag replicates, *n*), and the mean and standard deviation (SD) of the decomposition rate from the main text, from any tables and figures, and from the supplemental materials of the selected studies. If standard errors or 95% confidence intervals (CI) were given, we transformed them into SD values. If only figures were given, we used version 3.4 of the Webplot-digitizer software (https://automeris.io/WebPlotDigitizer/) to extract these parameters from the graphs. We also recorded the study location (longitude, latitude, elevation), climatic region (subarctic, boreal, temperate, subtropical, tropical, or other), ecosystem type (forest, grassland, shrubland, desert, wetland, stream, seagrass, lake, or ex situ microcosm), and litter substrate (leaf, root, stem, branch, straw, or other). Microcosm studies were further divided into two categories: terrestrial or aquatic. The former and latter, respectively, placed litter bags on the soil and in water. For litter substrate, most studies used terrestrial plants, but two studies used macrophytes for their decomposition experiment; these data were included in the main analysis and excluded from the subsequent analyses that focused on leaf litter. Note that authors reported the decomposition rate for mixed-species litter bags in different ways, as the values for all species together or for individual species in the same bag. Removing data based on the latter classification had little effect on our results, so we retained that data. Because of the limited data availability, we did not consider the potential influences of species richness (here, the number of litter species; more than 74% of the comparisons used two- or three-species mixtures) and instead considered species richness as a random term (see Data analyses). Based on the geographic locations of the studies, we estimated their present bioclimatic conditions based on the WorldClim database (www.worldclim.org).

### Species-mixing effects on litter decomposition

We calculated the unbiased standardized mean difference (Hedges’ *d*)^30^ of the decomposition rate between the mean values for the mixed- and mono-species litter. Hedges’ *d* is a bias-corrected, unit-free index that expresses the magnitude of the deviation from no difference in the response variable between comparisons. Note that many studies performed multiple comparisons for the decomposition rate between mixed- and mono-species litter, potentially causing issues of pseudo-replication and non-independence in the dataset, as is often the case for ecological syntheses^68^. We thus applied a multilevel random effects meta-analysis^30,69^ to account for this problem. In a random effects model, the effect sizes for individual comparisons are weighted by the sum of two values: the inverse of the within-study variance and the between-study variance. The multilevel model can also account for a nested structure in the dataset (in which different treatments and multiple comparisons were nested within each study) and is thus appropriate for dealing with non-independence within a dataset. To calculate the effect sizes, we defined their values to be positive for comparisons in which decomposition was faster in mixed-species litter than in mono-species litter and negative when mono-species litter decomposed faster. This is based on a species gain perspective. Note that some have mono- and two-species mixtures, and others could have for instance from 1 to 16 species in their experiments. Considering mono-species litter as a control is therefore the only way to be consistent across all studies. That is, quantification based on a species loss perspective requires us to obtain the effect sizes using mixed-species litter as a control. In this case, different studies have different levels of richness for a control, making it impossible to have a quantification in a standardized way. We first calculated the effect sizes based on mass loss and the decomposition rate constant *k* (Fig. 1b, c); because of the limited data availability for the constant *k*, we only calculated the effect sizes for the entire dataset and the subset of data for leaf litter. For mass loss, we further calculated the effect sizes for the different ecosystem types in different climatic regions; subsets of the data that originated from at least three different studies were used. We conducted a multilevel mixed effects meta-regression by considering random effects due to non-independence among some data points, resulting from a nested structure of the dataset that individual data points were nested within a treatment and treatments were also nested within a study. We used the *Q* statistic for the test of significance.

Note that, because publication bias is a problem in meta-analysis, we visually evaluated the possibility of such a potential bias by plotting the values of the effect sizes and their variances against sample size. If there is no publication bias, studies with small sample sizes should have an increased sampling error relative to those with larger sample sizes, and the variance should decrease with increasing sample size. In addition, the effect size should be independent of the sample size. Also, there should be large variation in effect sizes at the smallest sample sizes. Prior to the meta-analysis, we confirmed that these conditions existed (Supplementary Fig. 1), and found no publication bias large enough to invalidate our analysis.

We also visually confirmed that the datasets were normally distributed based on normal quantile plots. Furthermore, following the method of Gibson et al.^70^, we randomly selected only one comparison per treatment and then calculated the effect sizes for the decomposition rate (based on mass loss); we repeated this procedure 10,000 times (with replacement) and found that the species-mixing effects on decomposition were significantly positive (0.247 ± 0.045 for the mean ± 95% CI; Supplementary Fig. 2). We thus confirmed that the overall results were not affected by a publication bias or by non-independence of the dataset. We additionally focused on a subset of the entire dataset, which reported mass loss data for each component species from mixtures and thus had comparisons between mass loss of mono-species litter and that of the same species in a mixture (e.g., mass loss of mono-species litter for species A, B, and C was only compared with that for species A, B, and C within a three-species mixture, respectively). We found the results for this subset of the data (Supplementary Fig. 3) almost identical to the main results (Fig. 2a, b). Note that because of the limited data availability, we did not consider the effects of using different equation forms to estimate the rate constant *k* and instead considered these among-study differences based on a random term in our multilevel meta-analysis. We also calculated the influence of incubation period (days) on the effect size for the decomposition rate (based on mass loss of leaf litter) for the subsets of the dataset using a mixed effects meta-regression^30^ (Supplementary Fig. 4). We limited this analysis to studies that retrieved litter bags at least two different time points. To account for non-independence of data points, the study identity was included as a random term.

### Impacts of global change drivers on litter decomposition

Litter decomposition is primarily controlled by three factors: the environment (e.g., climate), the community of decomposers, and litter traits^46–48^. By relying on the dataset of Makkonen et al.^50^, who conducted a full reciprocal litter transplant experiment with 16 plant species that varied in their traits and origins (four forest sites from subarctic to tropics), we aimed to obtain a standardized equation to estimate how decomposition rate changed along a climatic gradient (hereafter, the “standardized climate–decomposition relationship”). First, by considering litter decomposition rates at the coldest location (i.e., the subarctic site) as a reference (control), we calculated the effect sizes (i.e., standardized mean difference based on Hedges’ *d*) for their decomposition rate constant *k* for all possible comparisons (i.e., between data from the coldest site and the value, one at a time, for the three other warmer sites). As explained above, the effect sizes were calculated only for the comparisons between decomposition rates of litter that were obtained using the same protocol (litter species, origin, and decomposers). The effect sizes therefore represent the climatic effects on litter decomposition after removing the effects of the decomposer community and litter traits.

We obtained the bioclimatic variables for these four study sites from the WorldClim database, and then modelled the standardized climate–decomposition relationships using a multilevel mixed effects meta-regression^30,69^. Annual mean temperature, the mean temperature of the wettest quarter, or precipitation of the driest quarter were used as an explanatory variable, with the protocol as a random term. This allowed us to evaluate how decomposition rate can be altered by climate along a latitudinal gradient from tropics to subarctic. We then calculated the decomposition rate constant *k* for our dataset in exactly the same manner as Makkonen et al.^50^ and calculated the effect sizes; because of limited datasets for most biomes, we performed this analysis only for the subset of our dataset that we obtained for forest biomes, and we used only leaf litter (57 studies) so the results would be comparable with those of Makkonen et al.^50^. We used these effect sizes and the standardized climate–decomposition relationships to convert the species-mixing effects into climatic effects; that is, based on the temperature or precipitation required to alter the effect size to the same magnitude as the litter diversity effect, we estimated the climate-equivalency of the diversity effects. This means that we relied on the slope of the relationships to quantify the diversity effects, as has been done in previous analyses^38,71^. We also compared these estimates to the future projections of changes in decomposition rates in response to climate change (with estimates for the 2070s for two of the representative concentration pathway scenarios of CO_2_ used in the 5th Climate Model Intercomparison Project; CMIP5 RCP 2.6 and 8.5) for the locations of the original studies (the coordinates of individual studies were collected using the Google Map; www.google.com). This comparison was aimed at quantifying the impacts of different global change drivers (biodiversity and climate change) on decomposition processes. Note that we additionally conducted the above analysis after excluding data with no species identity information for mono and/or mixed-species litter bags, those that measured ash-free dry mass loss (this exclusion was to be consistent with the procedure used in Makkonen et al.^50^), or both of them (Table S1). As an additional confirmation, we also relied on an alternative method used by Hooper et al.^45^ to convert the species-mixing effects into climatic effects (mean annual temperature), and compared the impacts of different global change drivers on the decomposition rate (Supplementary Fig. 5).

We further analyzed the potential changes in decomposition resulting from litter diversity and climate change. For the dataset of Makkonen et al.^50^, we first modelled the relationship between climate (annual mean temperature) and the decomposition rate constant *k* using an LMM with the protocol as a random term. The constant *k* was log-transformed to improve homoscedasticity. With this equation and the values of annual mean temperature at the 57 study locations in the forest biomes included in our dataset, we then estimated the expected values of *k* at these study locations (the present *k* values). Note that because the experiment of Makkonen et al.^50^ had no mixed-species litter, the expected *k* using the above LMM was used primarily to estimate the decomposition rate of mono-species litter at a given annual mean temperature. By combining this estimate with the aforementioned estimate of the climate-equivalency of the litter diversity effect (for annual mean temperature; Fig. 3a), it was possible to estimate the potential changes in the *k* that resulted from increasing litter diversity from mono- to mixed-species litter at the 57 forest study locations. Specifically, the diversity effect converted into the temperature effect (Fig. 3a) was added to present estimates of annual mean temperature at these study locations, and these values of temperature change were used as an explanatory variable to project changes in *k* based on the above LMM (the projected *k* values). In other words, we quantified the projected *k* values under a scenario in which we brought mono-species litter to areas with a warmer annual mean temperature equivalent to the temperature increase in the climate-equivalency effect. We then converted the present and projected values of the decomposition rate constant *k* to a mass loss per unit time, making it possible to calculate percentage changes in the decomposition rate due to litter diversification. Furthermore, using the above LMM for the temperature–decomposition relationship with the projected changes in mean annual temperature (based on the CMIP5 RCP 2.6 and 8.5 scenarios) as an explanatory variable, we projected future changes in the decomposition rate constant *k* at these study locations.

Again, we converted these estimates to a litter mass loss to obtain the percentage changes in the decomposition rate that would result from climate change. The rationale for using the temperature–decomposition relationship was as follows: A similar magnitude of increase in *k* can, in absolute terms, have different influences on decomposition rates at different locations with a different *k*. For instance, a change in the value of *k* from 0.1 to 0.2 and a change from 3.0 to 3.1 are substantially different when considered based on litter mass loss or litter amount remaining after decomposition. We thus included annual mean temperature in our model for quantifying the effects of litter diversity on decomposition.

## Supplementary information

Supplementary Information

Supplementary Data 1

Supplementary Data 2

## Data Availability

The effect sizes calculated for all comparisons are provided in Supplementary Data 2. Additional data that support the findings of this study are available from the corresponding author upon request.

## Code availability

The code that support the findings of this study are available from the corresponding author upon request.
