# Supplementary material for: A meta-analysis on decomposition quantifies afterlife effects of plant diversity as a global change driver
Source: Nat Commun. 2020 Sep 11;11:4547. doi: 10.1038/s41467-020-18296-w (PMC7486393; doi:10.1038/s41467-020-18296-w)
Supplement: Supplementary file 1 — Supplementary Information [file 41467_2020_18296_MOESM1_ESM.pdf]

Supplementary Information

A meta-analysis on decomposition quantifies afterlife effects of plant diversity as a global change driver

Mori et al.

This PDF file includes:

Supplementary Figure 1-6

Supplementary Table 1

Other Supplementary materials for this manuscript include the following:

Supplementary Data 1

Supplementary Data 2

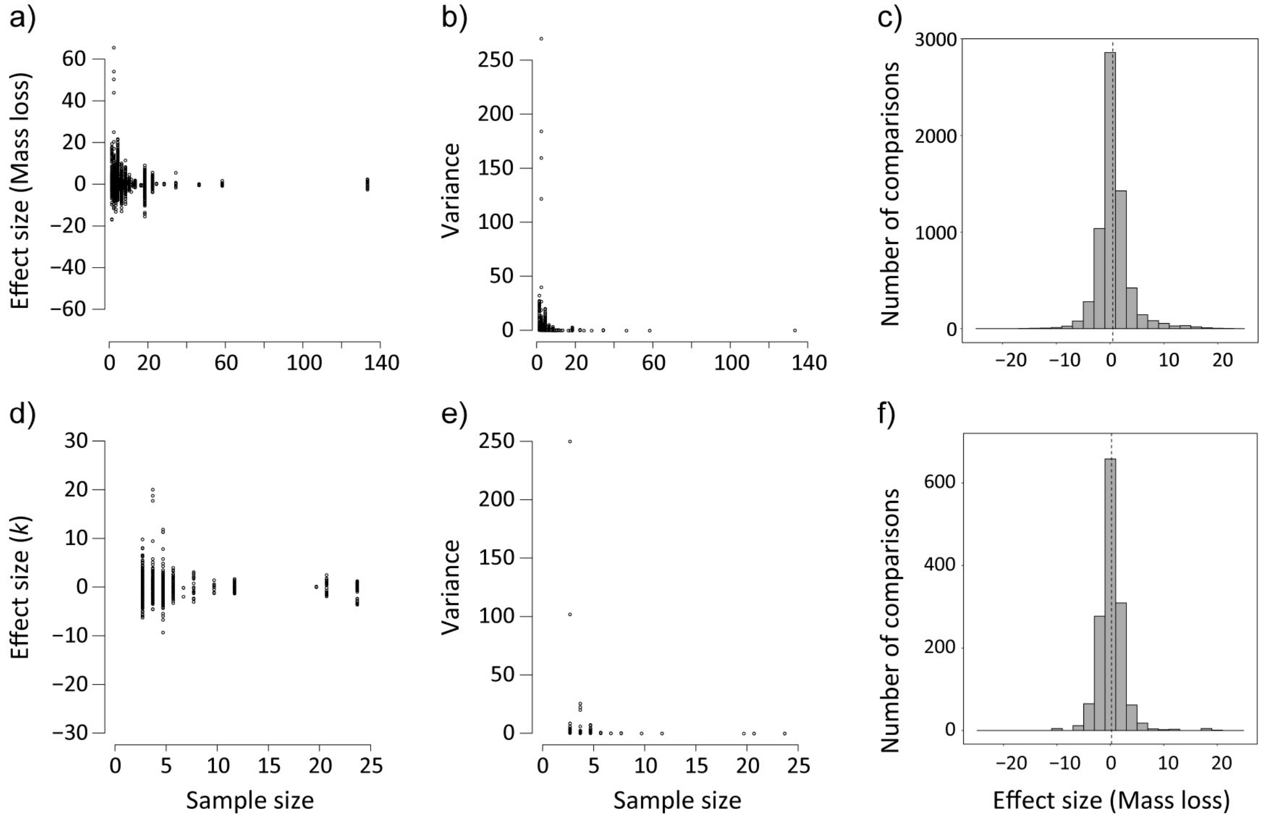

Supplementary Figure 1. The relationships between the sample size and the Hedges' *d* effect size and the variance of the litter decomposition rate based on mass loss (a-b) and the decomposition rate constant *k* (d-e). The frequency distribution of the Hedges' *d* effect size for the dataset based on mass loss estimation (c) and the decomposition constant *k* (d-e). The vertical dotted lines indicate mean values of the effect sizes. The dataset for mass loss measurement and estimation of the constant *k* comprises in total 6,535 comparisons across 1,949 different treatments reported in 131 studies and 1,423 comparisons across 504 different treatments reported in 45 studies, respectively. If there is no publication bias, studies with small sample sizes should have an increased sampling error relative to those with larger sample sizes, and the variance should decrease with increasing sample size. In addition, the effect size should be independent of the sample size, leading to no relationship between effect size and sample size. Finally, there should be larger variations in effect size at the smallest sample sizes. Here, these conditions were satisfied for the present meta-analyses, indicating that there was no publication bias large enough to affect our analysis.

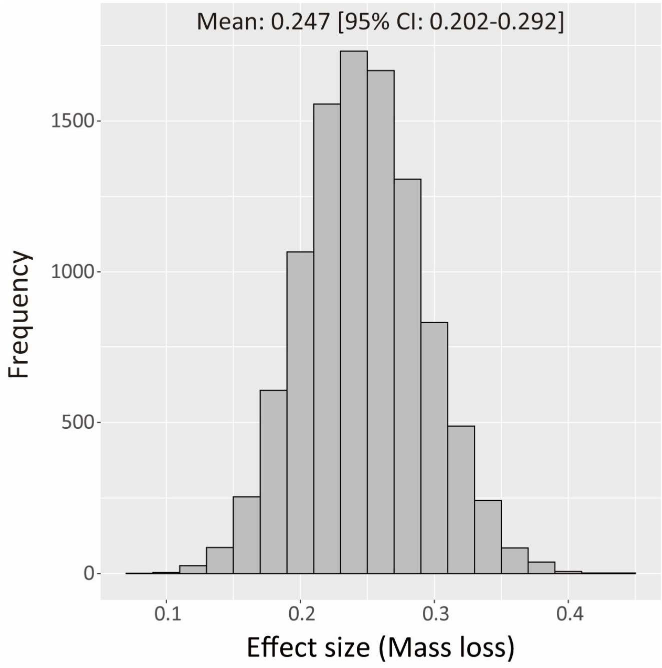

Supplementary Figure 2. Histogram showing the frequency distribution of the Hedges' *d* effect size. The effect sizes are for decomposition rate based on mass loss, calculated by resampling one comparison per treatment. Number of the treatment is 1,949. We repeated this resampling at 10,000 times (with replacement). Mean and the 95% CI (confidence interval) values are shown at the top. Overall, the result is equivalent to that based on the entire dataset (Fig. 1b; All), suggesting that the main results are little affected by autocorrelation and non-independence.

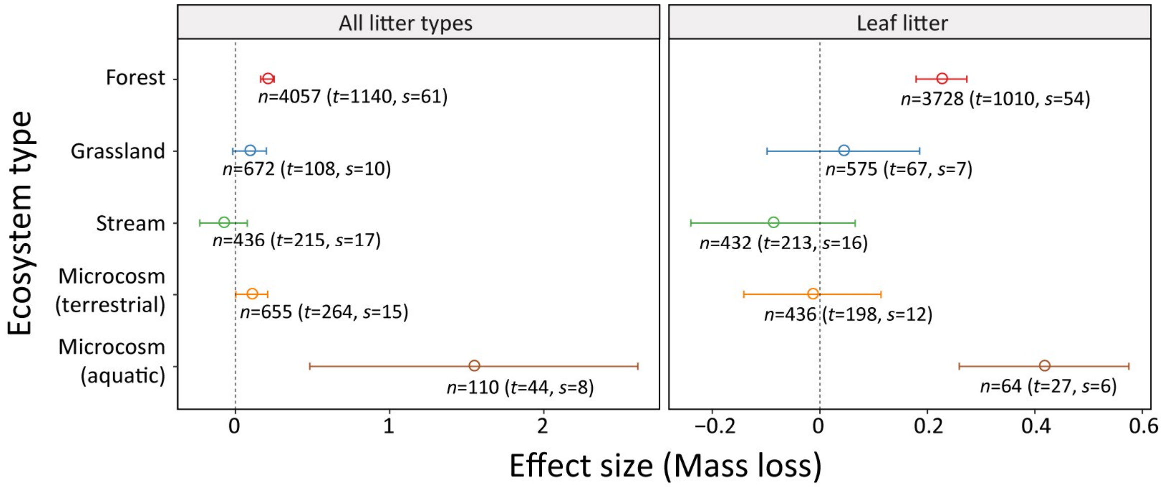

Supplementary Figure 3. The Hedge's *d* effect size for mass loss of all litter types and leaf litter analyzed by ecosystem types. The subsets of the entire data, which had litter species information for both mono and mixed-species litter bags, were used. Open circles and error bars represent means and the 95% confidence intervals, respectively. Number of comparisons (*n*), treatments (*t*), and studies (*s*) are shown. Positive values of the effect size indicate faster decomposition in mixed-species litter than mono-species litter. Vertical dotted lines are to indicate the effect size of zero.

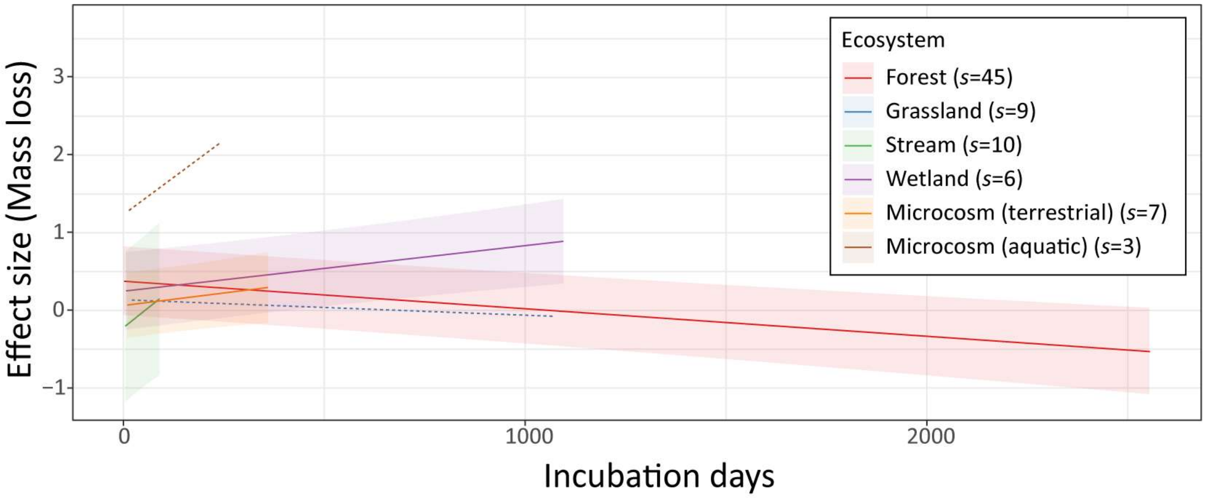

Supplementary Figure 4. The effects of number of days of incubation for litter bags on the Hedges' *d* effect size (for the decomposition rate based on mass loss). Solid lines and shaded areas indicate the fits and the 95% confidence intervals for the mixed effects meta regressions. For ecosystems that showed no significant change in the effect size with time, dotted lines are only shown.

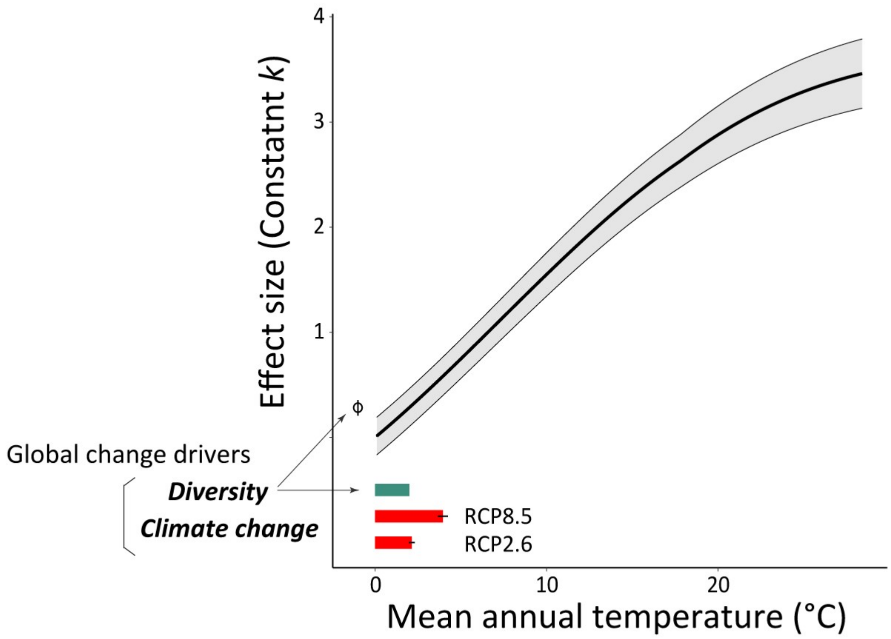

Supplementary Figure 5. Effects of global change drivers on litter decomposition. We calculated the Hedges' *d* effect size for the dataset of Makkonen, et al. [15], which was obtained from a full reciprocal litter transplant experiment that covered a wide range of biomes from the tropics to the subarctic, and applied the method of Hooper, et al. [18] to estimate the changes in decomposition rate as a function of the changes in mean annual temperature (shown as the solid black line and grey shaded area for the 95% confidence interval). By comparing the effect size obtained in our meta-analysis (shown as an open circle with an error bar for the 95% confidence interval), we estimated the temperature-equivalency (turquoise bar; the temperature required to increase the effect size of decomposition). In the study locations of our meta-analysis, we also obtained two future projections of temperature increases in the 2070s based on representative concentration pathways used in the 5th Climate Model Intercomparison Project, CMIP5 RCP 2.6 and 8.5; values are means (bars) and standard errors (whiskers). Overall, the litter diversity effects (species-mixing effects) on decomposition were comparable to the effects of future climate change.

PRISMA 2009 Flow Diagram

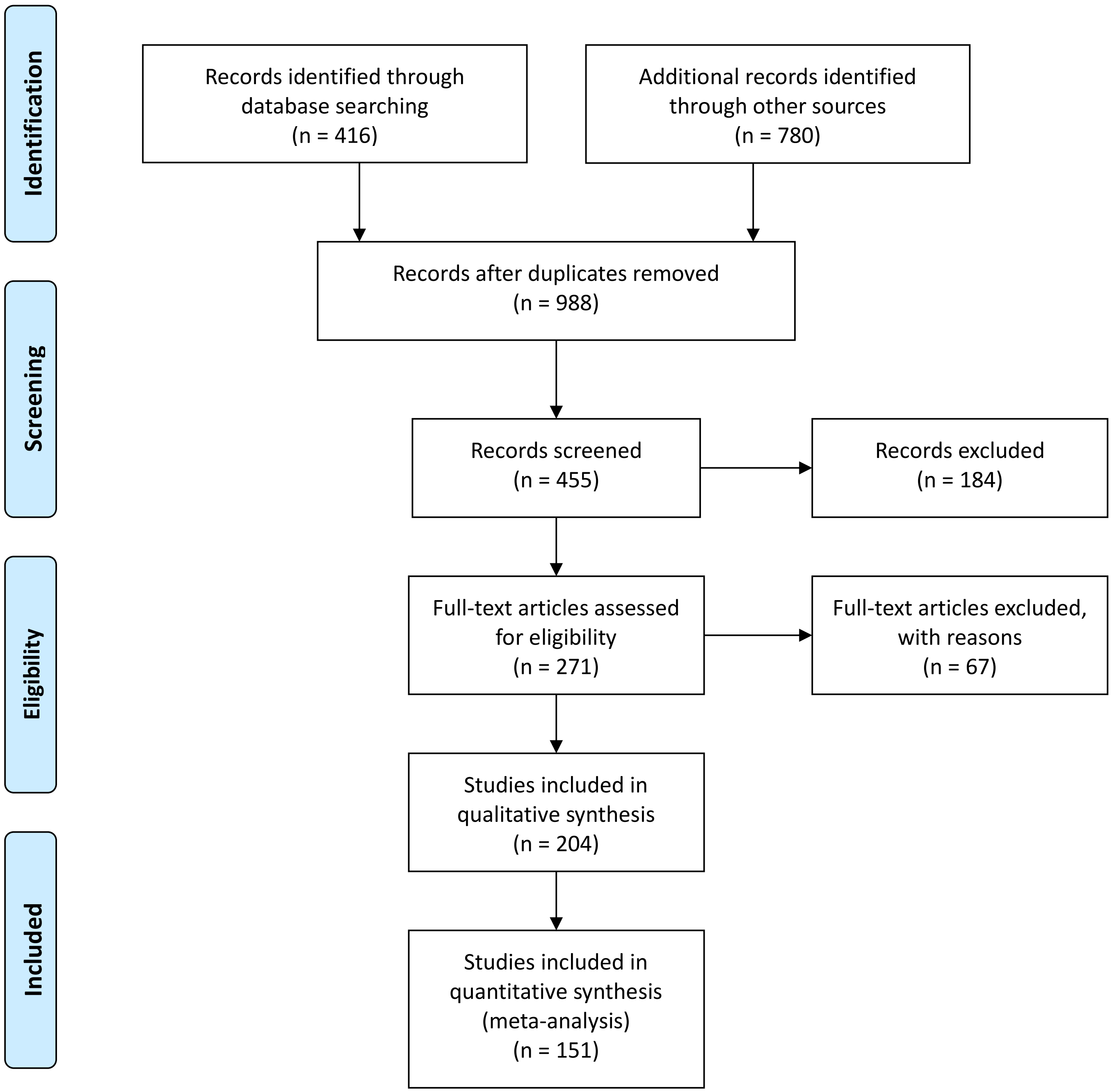

Supplementary Figure 6.

From: Moher D, Liberati A, Tetzlaff J, Altman DG, The PRISMA Group (2009). *P*referred *R*eporting *I*tems for *S*ystematic Reviews and *M*eta-*A*nalyses: The PRISMA Statement. PLoS Med 6(7): e1000097. doi:10.1371/journal.pmed1000097

For more information, visit www.prisma-statement.org.

Supplementary Table 1. Results of the climate-equivalency of the diversity effects using different subsets of the dataset. Number of comparisons (*n*), treatments (*t*), and studies (*s*) are shown.

Dataset	Climate-equivalency of the diversity effects (ºC)	n	t	s	Litter species		Measurement	
					Known	Unknown	Dry mass	Ash-free dry mass
Main result (Fig. 3a)	5.376	3,906	1,064	57	Yes	Yes	Yes	Yes
Subset1	5.702	3,697	1,010	54	Yes	No	Yes	Yes
Subset2	5.671	3,332	910	48	Yes	Yes	Yes	No
Subset3	5.989	3,184	864	45	Yes	No	Yes	No
Subset4	2.708	574	154	9	Yes	Yes	No	Yes
Subset5	3.038	513	146	9	Yes	No	No	Yes
